# Validating the Accuracy of a Patient-Facing Clinical Decision Support System in Predicting Lumbar Disc Herniation: Diagnostic Accuracy Study

**DOI:** 10.3390/diagnostics14171870

**Published:** 2024-08-26

**Authors:** Fatima Badahman, Mashael Alsobhi, Almaha Alzahrani, Mohamed Faisal Chevidikunnan, Ziyad Neamatallah, Abdullah Alqarni, Umar Alabasi, Ahmed Abduljabbar, Reem Basuodan, Fayaz Khan

**Affiliations:** 1 Department of Physical Therapy, Faculty of Medical Rehabilitation Sciences, King Abdulaziz University, Jeddah 22252, Saudi Arabia; fatimahassanb@gmail.com (F.B.); mgalsobhi@kau.edu.sa (M.A.); mfaisal@kau.edu.sa (M.F.C.); zneamatallah@kau.edu.sa (Z.N.); amalgarni@kau.edu.sa (A.A.); ualabasi@kau.edu.sa (U.A.); 2 Department of Physical Therapy, King Faisal Hospital, Makkah 24236, Saudi Arabia; aahmedalzahrani0035@stu.kau.edu.sa; 3 Department of Radiology, Faculty of Medicine, King Abdulaziz University, Jeddah 22252, Saudi Arabia; aabduljabar@kau.edu.sa; 4 Department of Rehabilitation Sciences, College of Health and Rehabilitation Sciences, Princess Nourah Bint Abdulrahman University, Riyadh 11671, Saudi Arabia; rmbasoudan@pnu.edu.sa

**Keywords:** artificial intelligence, machine learning, back pain, clinical decision support system, lumbar, disc herniation

## Abstract

Background: Low back pain (LBP) is a major cause of disability globally, and the diagnosis of LBP is challenging for clinicians. Objective: Using new software called Therapha, this study aimed to assess the accuracy level of artificial intelligence as a Clinical Decision Support System (CDSS) compared to MRI in predicting lumbar disc herniated patients. Methods: One hundred low back pain patients aged ≥18 years old were included in the study. The study was conducted in three stages. Firstly, a case series was conducted by matching MRI and Therapha diagnosis for 10 patients. Subsequently, Delphi methodology was employed to establish a clinical consensus. Finally, to determine the accuracy of the newly developed software, a cross-sectional study was undertaken involving 100 patients. Results: The software showed a significant diagnostic accuracy with the area under the curve in the ROC analysis determined as 0.84 with a sensitivity of 88% and a specificity of 80%. Conclusions: The study’s findings revealed that CDSS using Therapha has a reasonable level of efficacy, and this can be utilized clinically to acquire a faster and more accurate screening of patients with lumbar disc herniation.

## 1. Introduction

Low back pain (LBP) is a highly prevalent condition globally [1], with a prevalence rate of approximately 53.5% in Saudi Arabia [2]. LBP and its related disabilities are influenced by a combination of psychological, social, and biological factors, making it a complex disorder [3]. LBP can appear in different clinical and pathognomonic manifestations such as lumbar disc herniation and radiculopathy which are the two primary factors responsible for causing LBP [4]. However, lumbar disc herniation can be the cause of different types of pain which may lead to the overlap of dermatome and myotome, resulting in misdiagnosis of nerve root involvement [5]. Therefore, numerous clinical examinations have been conducted to determine the most accurate diagnostic test for detecting the likelihood and presence of radiculopathy caused by lumbar disc herniation. These examinations include straight leg raising, crossed straight leg raising, tendon reflexes, atrophy, and sensory deficits tests [6].

In addition, radiographic examination has traditionally been employed in primary healthcare settings and is broadly undertaken as a valid reference standard for identifying lumbar herniated discs [7]. Magnetic resonance imaging (MRI) and computed tomography (CT) have a considerable degree of accuracy in terms of specificity and sensitivity. The specificity of CT in detecting lumbar herniated discs with nerve root involvement ranges from 0.7 to 0.87, while its sensitivity ranges from 0.62 to 0.9. On the other hand, MRI has a specificity ranging from 0.43 to 0.97 and a sensitivity ranging from 0.6 to 1.0 in the same diagnosis [7]. However, the diagnosis of LBP is challenging for clinicians and requires a high level of skill, experience, and understanding of the complex physiological and anatomical structure of the human body [8].

A study done by Vining R et al. reported that only around 15% of surveyed LBP patients had accurate diagnoses with some degree of certainty [9]. Clinicians often gain diagnostic experience through an arduous, time-consuming process [8]. For example, understanding the human body’s complicated physiological and anatomical structure [8], a large patient load, and human limitations such as not being up-to-date on manual errors and the latest advancements [10]. Thus, the quality of medical care is frequently compromised, particularly in developing countries [11]. Therefore, this existing dilemma might be resolved by using CDSS to support the diagnosis of LBP and improve timely knowledge access [8].

Artificial intelligence (AI) will assist healthcare practitioners by providing reliable and expedient medical consultation services to improve the quality of treatment. It has the ability to supplement and enhance decision-making in various contexts of high importance [12,13,14]. CDSS can process patient information intelligently and present it to clinicians [15] or physiotherapists to support decision-making on the administration and assessment of various medical conditions such as assessing gait deviation in anterior cruciate ligament (ACL) injury [16], diagnosing and classifying peripheral neuropathy [17], predicting elderly quality of life [18], detecting coronary heart disease by using fuzzy rule-based system with multi-objective genetic algorithm [19] and helping clinicians in cancer treatment [20].

In the current study, researchers conducted a diagnostic accuracy study to assess the accuracy of an AI-enabled platform and an algorithm such as CDSS. The study examined a new web-based software called Therapha, which incorporates CDSS technology. This software aims to assist clinicians in diagnosing LBP by providing them with real-time support and guidance. Therapha is a triaging software that guides the clinician through a comprehensive patient history consisting of medical history, social history, family medical history, self-reported pain evaluation, functional deficits, psychological issues, sleep patterns, and posture. Upon concluding the patient history, it generates the most likely differential diagnosis related to the LBP. This emerging technology has the potential to serve as a valuable instrument for medical healthcare professionals in the process of making clinical decisions [21,22]. Consequently, it has the potential to enhance both the accuracy of diagnoses and the promptness of accessing knowledge [8]. The objective of this study was to assess the diagnostic precision of AI as a CDSS in comparison to MRI for diagnosing patients with lumbar disc herniation.

## 2. Materials and Methods

### 2.1. Study Design

The study was designed in three stages: (i) a case series was conducted to generate hypotheses and examine the effectiveness of a novel patient-facing assessment; (ii) the Delphi method was used to validate the prediction accuracy of Therapha and evaluate the capability of the CDSS in comparison to experts’ opinions; (iii) a diagnostic accuracy study was conducted to assess the prediction accuracy of Therapha web-based software in contrast to MRI in diagnosing lumbar radiculopathy.

### 2.2. Participants

The participants were male and female patients over the age of 18 complaining of LBP with radiating symptoms extending down to the leg. Patients who were suffering from any other LBP pathology, history of cancer, previous spine surgery and failed back syndrome were excluded. Before obtaining the patients’ clinical and personal background, all participants provided their signature on a written informed consent document. The ethical approval was obtained from the research ethics committee at King Abdulaziz University Hospital (KAUH) (HA-02-J-008), Jeddah, Saudi Arabia.

### 2.3. Case Series

**Figure 1:**
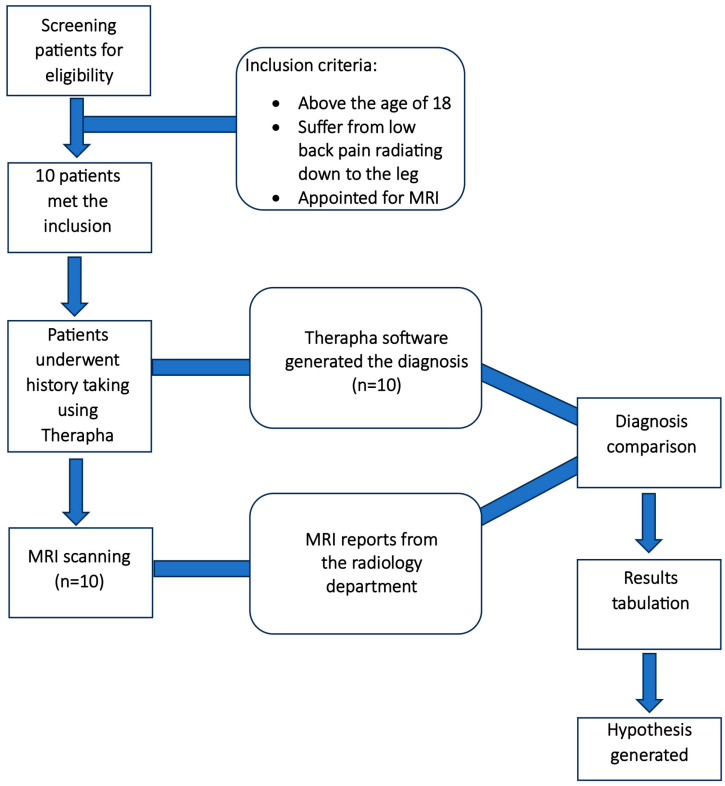
Design of case series to generate the study hypothesis.

The current study began with a preliminary case series to determine the Therapha web software’s accuracy in diagnosing patients with herniation of the lumbar disc. This study comprised 10 patients with LBP who met the inclusion criteria. All patients underwent an MRI, and the patients’ history was collected by a blind assessor using Therapha web software on the same day before the MRI procedure. Therapha analyzed the patient data and generated the most likely differential diagnosis of the LBP. A study assistant with 7 years’ experience of working in the field of radiology matched the software’s final report to the MRI report and tabulated the data (Therapha and MRI final diagnosis) for each patient (Figure 1).

### 2.4. Delphi Method

**Figure 2:**
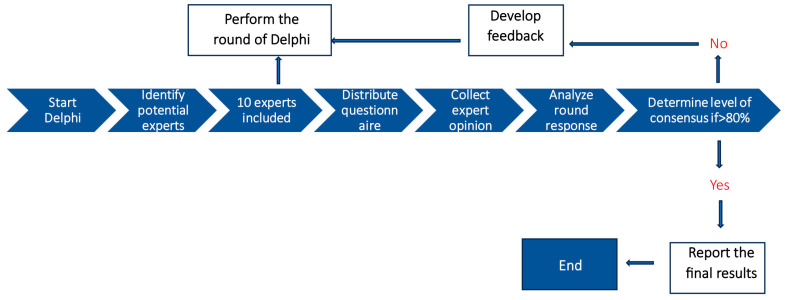
Delphi study steps for Therapha software validation on patients with low back pain.

The Delphi method has been used in various medical and healthcare settings [23,24] to determine expert consensus in situations where there is insufficient evidence available [25]. The Delphi method is an effective process that uses multiple rounds of expert voting, in which a questionnaire is distributed to a group of experts who respond anonymously to the questions. The survey results are then arranged in a tabular form and reported back to the experts, and each panel is requested to complete the questionnaire once again [24] (Figure 2).

#### 2.4.1. Panel Selection

A total of ten experts have participated in this study to validate Therapha software. The panel was chosen based on their research and clinical experience in assessing and treating patients with LBP. Experts were from multiple countries including Saudi Arabia, India, the United Arab Emirates, the United Kingdom, and the United States.

#### 2.4.2. Round 1

In the first round, all experts were emailed a set of statements, instructions for rating, and an explanation of the study aims. Experts were asked to read the patient clinical history summaries that were automated by the Therapha software from subjective reporting and were asked to give a final diagnosis for each patient. Then, a rating was made on the experts’ final diagnosis (agree/disagree with the Therapha diagnosis) on a scale of 1 to 9, where a score of 1 to 3 score was classified as “inappropriate”, 4 to 6 as “uncertain”, and those in the range of 7 to 9 as “appropriate” [24]. Panelists were asked to justify their vote if they chose to vote for “disagreement”.

Therapha prediction was consolidated with an 80% agreement among the experts (8 out of 10 panelists) to accept or remove a statement during the construction of the final diagnosis. Based on Lynn’s et. al. findings, 80% was determined as an adequate cut-off [26]. Statements that did not receive 80% agreement were amended based on feedback from the expert panel and circulated to panelists in the second round.

### 2.5. Diagnostic Accuracy

#### 2.5.1. Sample Size

The sample size was calculated by using G*power software (version 3.1.9.7, Dusseldorf, Germany), with an alpha of 0.05, a power of 0.8 and an effect size of 0.5. Based on the software calculation, the minimum required number was 90.

#### 2.5.2. Assessment

Upon receiving the patient’s consent, their personal and clinical history were taken using Therapha software on the same day before the patient underwent an MRI. Each software-assisted patient evaluation session lasted around 10–15 min. The history encompassed initial demographic details and questions about the patient’s medical history, family history, social history, functional deficits, the severity of the back pain, nature of the pain, duration, onset, location, easing and aggravating factors, abnormal sensations, and the mechanism of injury and its relationship to the emergence of pain. Also, patients were asked about symptoms that may indicate systemic disease infection, fracture, or neurological impairments.

#### 2.5.3. Procedure

**Figure 3:**
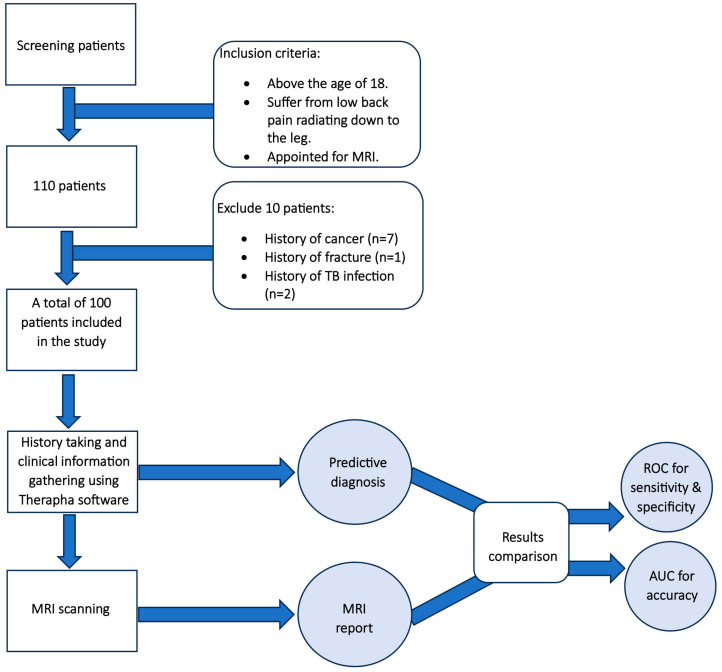
Flowchart demonstrating the diagnostic accuracy study process of the Therapha software.

Patients were referred to the Radiology department from orthopedic, neurology, and spine surgery clinics. The Therapha software is only available in English, hence requiring the use of a blind assessor. An experienced physical therapist, who was blinded to the patient’s information, utilized Therapha software to gather the patient’s personal and clinical history before the MRI. The final report received from the software was compared to the gold standard (MRI reports) by the study assistant and excluded any other pathology such as infection, fracture or tumor (Figure 3).

### 2.6. Outcome Measures

#### 2.6.1. Therapha Software

Therapha (v1.3.5) web software is a CDSS that is considered a comprehensive screening tool and triaging system for spine pathology which supports self-service by the patient or clinician. This system utilizes the power of information technology to identify medically established patterns by incorporating over 4000 independent variables, which is evidently superior to the clinical reasoning by a human clinician. This algorithmic approach enables the tool to predict the possible differential diagnoses in an order of probability percentile. The system divides spine disorders into subgroups besides conventional classification. The supervised AI model identifies patterns in the same manner as a clinician conducts an evaluation, and the Therapha system is capable of modifying their algorithm in the light of newer research findings. This improves the solution’s predictive accuracy over the period through an iterative process. CDSS uses data from the clinician or patient self-administered questionnaire to refine clinical reasoning and predictions.

The information gathered from the patient generates a ‘digital twin’, which serves as the primary input to this clinical decision support solution. The other main component is the knowledge repository, which utilizes the data captured by the chatbot to generate the top three hypothetical diagnoses. The algorithm is the software that embodies AI, which is the system’s most innovative component.

#### 2.6.2. MRI

In this study, all patients were scanned with MRI, using 3 Tesla Verio and Skyra systems (Siemens, Erlangen, Germany). A slice thickness of 3 mm disc by disc was used with an interslice gap of 1–0.5 mm.

### 2.7. Statistical Analysis

Data were statistically analyzed by using SPSS for Windows (version 23.0; SPSS Inc, Chicago, IL, USA) to evaluate the predictive accuracy of Therapha web software with MRI reports. Data were analyzed for normality using the Shapiro–Wilk and Kolmogorov–Smirnov tests. Anthropometric data were analyzed using descriptive analysis. The accuracy of the software performance was measured using the Receiver Operating Curve (ROC) to find the Sensitivity and Specificity. The ROC curve was plotted by True Positive Rate (TPR)/False Positive Rate (FPR), where TPR or Sensitivity is expressed as TPR = TP/(TP + FN), where TP is the number of true positive instances, and FN is the number of false negative instances. The FPR or Specificity is calculated as FP/FP + TN, where FP is the number of false positives and TN is the number of true negatives. *p*-value < 0.05 was considered statistically significant.

## 3. Results

### 3.1. Case Series

Lumbar disc herniation was verified by MRI for all patients (*n* = 10) except for cases # (4 and 6) who had a different spine diagnosis by AI software. Patients’ Therapha software inferences and MRI reports are illustrated in Table 1.

MRI findings for case# 4 showed “no significant disc disease”; however, Therapha identified it as “Lumbar Radiculopathy/Lumbar Disc Herniation/Lumbago with Sciatica”. This can be explained by the nature of the pain. As the patient fed into the chatbot, they reported dull and radiating pain from the back spreading to the L3-L4 dermatomal regions, along with tingling and numbness covering the lateral aspect of the thigh and lower leg corresponding to the L4-S1 dermatomes. There was also tenderness in the midline corresponding to the L4 segment. Furthermore, the patient mentioned trouble with functional activities such as squatting, climbing stairs, and walking, and also reported decreased intolerance to activities like bending forward, standing, and sitting. As a result, patients complain of restriction in daily living activities and notice guarding and limited movement of the involved areas.

Regarding case# 6, the MRI report was “extradural meningeal cyst”, while Therapha predicted the patient as “Lumbar Radiculopathy/Lumbar Disc Herniation/Lumbago with Sciatica”. Researchers can interpret this from the patient’s medical history since the patient reported his/her pain as a dull ache, radiating in nature, and marked the corresponding area to T12-L1 dermatomal distribution. The patient also had abnormal sensations of tingling and numbness in the feet in L_4_-S_1_ dermatomal regions and the presence of tender spots. In addition, the patient’s pain worsened in the morning and got better as the day progressed. Also, the pain was exacerbated by deep breathing, laughing, sneezing or coughing. The patient reported difficulty in moving the affected area, as well as performing functional activities like squatting, climbing stairs, and walking. The complaints noted by the patient were aggravated with standing, walking, and sustained positions. Moreover, the patient reported a decreased tolerance in activities such as coming up from a bent-over position and from sitting to standing positions, bending forward, standing, and sitting. As a consequence, the patient complained of restriction in daily living activities and noticed guarding or limiting movement of the involved area. Furthermore, the patient had a history of falling. The patient reported an easing of their symptoms with rest in the supine position and medications.

### 3.2. Delphi Method

Following the completion of the case series, the assessor collected and tabulated the experts’ rating sheets. In the first round, all respondents agreed (*n* = 10) that the patients’ medical histories were congruent with Therapha’s prediction, thereby achieving consensus. As all 10 cases had a consensus of above 80%, there was no need to re-circulate rating sheets for a second round.

Regarding cases# (2, 3, 4, 5, 8, 9, and 10), there was 100% consensus that the patients’ medical histories were consistent with Therapha predictions. However, regarding cases #1, #6, and #7, there was a consensus of 90% among all 10 experts.

### 3.3. Diagnostic Predictive Accuracy

**Figure 4:**
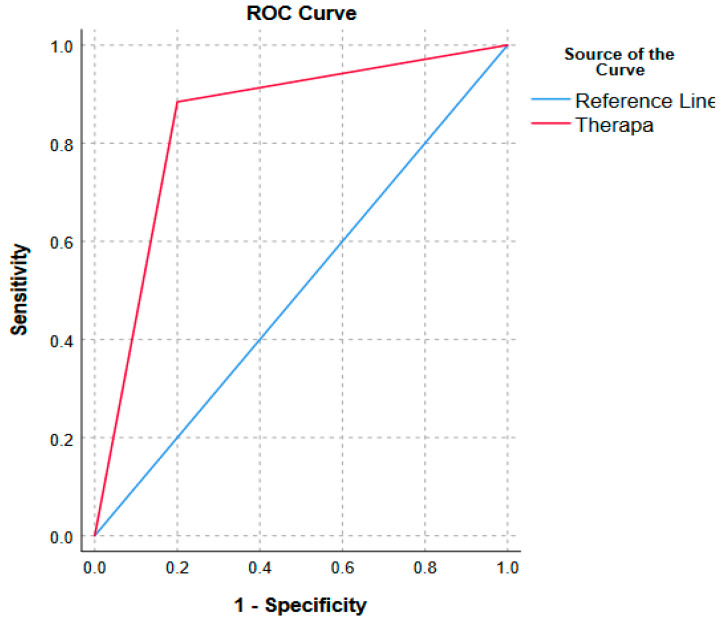
Receiver Operating Curve (ROC).

The demographic characteristics of patients who participated in this study are displayed in Table 2. The detailed clinical and MRI data of the 100 patients who were included in the diagnostic study are described in Supplementary Material Table S1. The accuracy of predictions from the software was determined by using ROC. The curve was up toward the left with an area under the curve of 0.84 (*p* = 0.001, 95% CI; 0.6 to 1.0) (Figure 4). Therapha had a sensitivity of 88%, specificity of 80%, positive predictive value of 99%, negative predictive value of 27%, positive likelihood ratio of 4.4, and negative likelihood ratio of 0.15.

## 4. Discussion

In this study, a novel web software has been proposed for predicting lumbar disc herniation. The results of this study showed that Therapha software can be used for predicting and assisting with the diagnosis of lumbar disc herniation with a high level of accuracy. Findings revealed that Therapha technology has a satisfactory level of effectiveness, making it suitable for expedited and more effective screening of patients with lumbar disc herniation. To the best of our knowledge, this is the first study to examine the efficacy of an AI-enabled tool compared to the gold standard (MRI) for diagnosing patients with lumbar disc herniation.

### 4.1. Delphi Method

The Delphi method was used to reach an agreement in diagnosing patients with lumbar herniated discs. In the present study, experts agreed on statements representing the final diagnosis of patients that were generated by Therapha software which can be used as a guideline for clinical practice to help in decision-making process. This reinstates the significance of the tool’s potential to be used as an adjunct to provide care that is backed by the power of technology.

Authors began with the patient history collection, ensuring a comprehensive and unbiased approach, and subsequently moved to analyzing the MRI results in detail. This methodological sequence, which kept the assessor blind as to whether patients had lumbar disc prolapse or a different pathology, was designed to mitigate any potential biases from the assessor during the initial phase (patient history-taking). 

Each patient’s signs and symptoms were in match to their MRI findings. Since most patients had the same history of lumbar herniated disc, patient compliance with LBP accompanied by radiculopathy and abnormal sensations like numbness and tingling were reported. Most patients in this study showed an increase in their symptoms with flexion activities which eased with extension-based movements. The majority of patients complained of pain during prolonged activities such as standing, sitting, or even walking for a long time. Also, patients reported that sneezing, deep breathing, or coughing exacerbated their symptoms. In addition, patients had the same type of pain “dull, aching, radiating to the lateral side down to the leg and foot”. Furthermore, muscle spasms and weakness of either the back or thigh muscles were reported.

Concerning cases # (4 and 6), the Therapha predictions aligned with the expert diagnostic findings. However, the experts’ agreements were not entirely consistent with the results obtained from the MRI scans. They had a consensus of 100% and 90% for cases # (4 and 6), respectively. Based on the findings, it could be stated that Therapha can identify patients with lumbar disc herniation efficiently which could help clinicians in their decision-making process by serving as a cognitive aid.

Therapha software is an innovative tool that can assist healthcare professionals in analyzing patient history (screening and diagnostic questions) and provide the most likely differential diagnosis without disturbing the clinical workflow. The solution accomplishes this in an innovative way by replacing the traditional patient intake forms with a conversational chatbot. The entire interaction is automated into an organized clinical summary and provides an actionable, insightful report that includes probable differential diagnoses. Screening questions help to identify patients who require special or alternative care pathways [23] while diagnostic questions help to identify the proper pathology, allowing for suitable treatment selections [23].

The use of proper diagnostic and screening questions can improve patient care by successfully identifying problems and discovering diseases correctly [23]. History-taking alone has been proven to accurately identify clinical problems 56% to 82.5% of the time [27,28]. Therapha, however, has an accuracy of 84% in predicting lumbar disc herniation pathology through history-taking incorporated with AI-based technology.

However, this procedure does not diminish the significance of physical examination. To confirm a diagnosis, a physical examination should be done in conjunction with a history. Physical examinations can sometimes reveal unexpected diagnoses or contribute to previously obtained data [29]. Investigations can also help in LBP diagnosis; medical imaging advances, in particular, have offered healthcare professionals new, non-invasive approaches to improve patient care [30].

### 4.2. Diagnostic Predictive Accuracy

Various systems have been developed to assist in diagnosing different pathologies. However, there are limited systems in the literature that are specifically designed to help in the diagnosis and treatment of LBP [31,32,33]. Although AI-based solutions have been developed in a variety of medical disciplines, research incorporating AI in LBP is still in its embryonic stage [34].

The result of this study was in line with Kadhim et al. who studied a fuzzy expert system for diagnosing LBP patients depending on medical observation symptoms detected using the fuzzy system and other factors that may influence back pain diagnoses such as body mass index, gender, age, and patient history. Thereafter, depending on the information provided, this system offered the most likely spine pathology and treatment. The system showed an accuracy of 90% when compared to other specialist diagnoses [35].

Furthermore, Lin et al. examined a web-based decision support system that used production for knowledge representation to evaluate a patient’s information and suggest a diagnosis for LBP. The system infers multi-part diagnoses rapidly using a mini-Bayesian approach. It includes two interfaces for knowledge update and convenient system access, an inference engine, a knowledge base and a case repository. The CDSS shows performance equal to that of human experts [8]. However, this web-based decision support system needs to expand on its knowledge replenishment support. A clinician’s diagnostic knowledge is likely to accumulate over time, making the system’s knowledge replenishment support important. On the other hand, Therapha web software is currently built on a hybrid algorithm that utilizes over 4000 independent variables and is periodically updated depending on feedback from clinicians on the predictions made. They verify and discuss the feedback from clinicians and then make the required changes to improve the algorithmic power. The vision is to create a learning system as the system absorbs large quantities of clinical data over the course of time.

Sari et al. examined two expert systems (adaptive neuro-fuzzy inference system and artificial neural network) to objectively assess the level of pain intensity in low back pain patients. Researchers used two input variables including skin resistance, a visual analog scale, and one output variable which was LBP intensity [36]. They found higher accuracy in predicting LBP when the two systems were used in combination. Sari et al. proposed an objective and subjective method that can be used to scale a patient’s level of pain [36]. However, this cannot be used as a diagnostic tool, rather it can be used as a screening tool. On the other hand, the current study presents a clinical decision support tool (Therapha) which is part of its algorithm to determine LBP intensity subjectively by using a visual analog scale and detailed history related to pain.

In recent years, the area of AI has been growing rapidly, with multiple studies demonstrating high levels of accuracy in various AI applications. Indeed, a growing body of literature has reported the high level of accuracy of AI systems in various medical fields, especially in medical imaging. Lee et al. converted lumbar CT scans into axial T2-weighted MRI slices by using generative adversarial networks. Then, they compared them to each patient’s actual lumbar MRI images [14]. The results show an 80.2% similarity to the actual lumbar MRI images. Moreover, Ramirez et al. used AI techniques such as decision trees, support vector machines, and a logistic regression classifier to identify scoliosis curve changes. They found that the support vector machine generated the best results on 141 radiological spine scans, with an accuracy of 86%, and applied AI on MRI scans to estimate the surgical level of patients having disc decompression surgery [37,38]. Harada et al. recently developed an algorithm that identified patients at risk of re-herniation after microdiscectomy. After validation, the prediction method showed a recall of 80%, an area under the ROC of 72%, and an accuracy of 70% [39].

The above-mentioned studies showed high accuracy and performance, as Therapha software has shown. However, Therapha is a triaging system assessing physical problems related to the musculoskeletal system, but it does not assess and read the MRI itself. Image processing and computer vision are gaining momentum as computational resources such as powerful graphics processing units become more readily accessible. Indeed, current spine research studies using AI methods are focused on medical imaging. However, AI research in other areas such as spine pathology diagnosis is lacking, and more studies and research are required.

A systematic review examined the effectiveness of neurological tests, including sensory, motor, reflex, and neuro-dynamic assessments, in diagnosing lumbo-sacral radiculopathy by comparing them to MRI, electro-diagnostic tests, or intra-operative findings. The findings indicated that the diagnostic accuracy of most clinical neurological tests is generally low to moderate. There appears to be a lack of consensus among researchers regarding the diagnostic value of lower limb neuro-dynamic tests [40]. It remains unclear whether these tests can reliably identify disc herniation and subsequent nerve root compression, or if they are more indicative of hypersensitivity in the sacral and femoral plexus due to mechanical irritation [40].

Further, a systematic review and meta-analysis assessed the diagnostic effectiveness of sensory, motor, and reflex tests for identifying disc herniation and radiculopathy. The review compared neurological test results with surgical and radiological reference standards for disc herniation, and uniquely examined the connection between neurological findings and specific levels of herniation. The overall findings indicated that the diagnostic accuracy of all components of the neurological examination in detecting disc herniation in patients with suspected radiculopathy was limited. This was consistent regardless of the testing procedure or the specific level of herniation. The tests demonstrated poor diagnostic accuracy, with low sensitivity, moderate specificity, and limited overall accuracy, independent of the reference standard or herniation level [5]. Conversely, Therapha showed high sensitivity compared to the gold standard MRI. Therapha’s approach not only assesses patient signs and symptoms without aggravating pain but also includes a comprehensive patient history, covering medical history, social history, family medical history, self-reported pain evaluation, functional deficits, psychological issues, sleep patterns, and posture [5].

This study had several limitations. The software was only used for the lumbar area to identify patients with radiculopathy, excluding other conditions or lesions associated with back pain. Additionally, although the software was intended to detect various pathologies connected to lower back pain, it did not encompass all possible conditions. Furthermore, the study exhibited a generalization bias by exclusively recruiting individuals with symptoms related to the lower back and lower limbs, which accounts for the lower negative predictive value (NPV) of 27%. Also, the age group of the patients in the study is worth mentioning; it is noteworthy to state the age group of the participants involved in the study as a limitation to generalizing the findings.

In addition, the study had no control over the MRI devices, radiology technicians, and radiology consultants. Another limitation of the study was that the software is only available in the English language. As mentioned before, Therapha software supports patient self-service. However, the majority of the patients in this study did not understand English, so translation to other languages is important in order to increase access to the patient self-service features in the software. In this study, a patient’s history was taken and entered into the software by the clinician. Further, this study had several strengths, the software is a cost-effective and quick screening tool that may aid patients who are contraindicated to MRI such as claustrophobic individuals. For future research, it is recommended to investigate and study other spinal conditions such as cervical, thoracic, sacroiliac, and other conditions related to the lumbar region.

## 5. Conclusions

Given the increasing expenses associated with diagnosing and treating LBP, Therapha could be a highly cost-efficient tool to improve diagnostic accuracy and deliver effective care. Therapha’s patient-centered approach utilizing AI to augment decision-making will lead to enhanced clinical outcomes in spine care. This study provides new insights for healthcare researchers, practitioners, and policymakers into the potential of implementing emerging technologies, especially AI, in healthcare delivery and diagnosis. As evidenced in the study, CDSS can be an efficient cognitive support for clinicians to render optimal patient care. Future studies are required to investigate additional spinal illnesses such as sacroiliac, facet joint disorders, etc., not limited to the lumbar region but to the thoracic, and cervical segments as well.

## Figures and Tables

**Table 1:** MRI findings and Therapha software inferences.

Patient No.	Age	BMI	MRI Diagnosis	THERAPHA Prediction
1	55	38	Mild diffuse disc bulge	Lumbar radiculopathy/Lumbar disc herniation/Lumbago with Sciatica
2	67	26	At the level of T12-L1 there is mild central disc bulge	Lumbar facet joint pain/lumbar zygapophyseal/non-specific low back pain
3	59	22	Mild diffuse disc bulge	Lumbar radiculopathy/lumbar disc herniation/lumbago with sciatica
4	40	21	No significant disc disease	Lumbar radiculopathy/lumbar disc herniation/lumbago with sciatica
5	33	25	Diffuse posterior bulge	Lumbar radiculopathy/lumbar disc herniation/lumbago with sciatica
6	38	25	Extradural meningeal cyst	Lumbar radiculopathy/lumbar disc herniation/lumbago with sciatica
7	65	32	Minimal diffuse posterior disc bulges	Lumbar radiculopathy/lumbar disc herniation/lumbago with sciatica
8	61	34	Lumbar disc bulge L4-5, L5-S1	Lumbar radiculopathy/lumbar disc herniation/lumbago with sciatica
9	40	29	Lumbar disc bulge L4-5, L5-S1	Lumbar radiculopathy/lumbar disc herniation/lumbago with sciatica
10	69	25	Lumbar disc bulge L4-5, L5-S1	Lumbar radiculopathy/lumbar disc herniation/lumbago with sciatica

**Table 2:** Descriptive statistics (*n* = 100).

	Female		Male	
	Mean ± SD	Median (MinߝMax)	Mean ± SD	Median (MinߝMax)
Age (Years)	48 ± 14	48	48.5 ± 13.3	50
Height (cm)	158.8 ± 5	159	173 ± 6	170.5
Weight (kg)	75.3 ± 15	72	82 ± 12.6	80
BMI	29.7 ± 6	29	27.6 ± 3.9	26

## Data Availability

The data presented in this study are available on request from the corresponding author. The data are not publicly available due to privacy restrictions.

## Supplementary Materials

The following supporting information can be downloaded at: https://www.mdpi.com/article/10.3390/diagnostics14171870/s1, Table S1: MRI findings and Therapha software inferences of patients in the Diagnostic Accuracy study.
