# Supplementary material for: Validating the Accuracy of a Patient-Facing Clinical Decision Support System in Predicting Lumbar Disc Herniation: Diagnostic Accuracy Study
Source: Diagnostics (Basel). 2024 Aug 26;14(17):1870. doi: 10.3390/diagnostics14171870 (PMC11394625; doi:10.3390/diagnostics14171870)
Supplement: Supplementary file 1 [file diagnostics-14-01870-s001.zip › diagnostics-3125743-supplementary.pdf]

Patient no.	Age	BMI	MRI Diagnosis	THERAPHA Prediction
1	58	25	lumber disc bulge L5-S1	Acute (Or) Subacute (Or) Chronic Low Back Pain With Radiating Pain
2	40	26	lumber disc bulge L5-S1	Acute (Or) Subacute (Or) Chronic Low Back Pain With Radiating Pain
3	57	24	lumber disc bulge L2-L3, L3-L4, L4-L5, L5-S3	Acute (Or) Subacute (Or) Chronic Low Back Pain With Radiating Pain
4	84	26	lumber disc bulge L5-S1	Lumbar Radiculopathy/Lumbar Disc Herniation/ Lumbago With Sciatica
5	18	28	Low back pain - clear	Spinal Instabilities (Dynamic Or Passive), Lumbar Region
6	30	29	lumber disc bulge L5-S1	Lumbar Radiculopathy/Lumbar Disc Herniation/ Lumbago With Sciatica
7	53	29	lumber disc bulge L4-L5, L5-S1	Lumbar Intervertebral Disc Disorder With Radiculopathy
8	24	21	lumber disc bulge L4-L5, L5-S1	Lumbar Radiculopathy/Lumbar Disc Herniation/ Lumbago With Sciatica
9	40	28	lumber disc bulge L4-L5, L5-S1	Lumbar Radiculopathy/Lumbar Disc Herniation/ Lumbago With Sciatica
10	45	30	lumber disc bulge L4-L5, L5-S1	Lumbar Intervertebral Disc Disorder With Radiculopathy
11	45	38	lumber disc bulge L4-L5, L5-S1	Lumbar Discogenic Disease
12	60	33	lumber disc bulge L4-L5, L5-S1	Lumbar Radiculopathy/Lumbar Disc Herniation/ Lumbago With Sciatica
13	23	21	Low back pain - clear	Spinal Instabilities (Dynamic Or Passive), Lumbar Region
14	35	31	Low back pain, ms. spasm - clear	Degenerative Disc Disease, Lower Back/ Degenerative Joint Disease, Lumbar Spine/ Lumbar Spondylosis/Lumbago
15	44	35	L5-S1 spondylosis	Lumbar Intervertebral Disc Disorder With Radiculopathy
16	44	29	lumber disc bulge L4-5, L5-S1	Lumbar Radiculopathy/Lumbar Disc Herniation/ Lumbago With Sciatica
17	23	23	lumber disc bulge L5-S1	Lumbar Radiculopathy/Lumbar Disc Herniation/ Lumbago With Sciatica
18	66	36	lumber disc bulge L3-4, L4-5, L5-S2	Lumbar Radiculopathy/Lumbar Disc Herniation/ Lumbago With Sciatica
19	60	29	lumber disc bulge L5-S1	Lumbar Intervertebral Disc Disorder With Radiculopathy
20	34	24	lumber disc bulge L4-5, L5-S1	Lumbar Radiculopathy/Lumbar Disc Herniation/ Lumbago With Sciatica
21	83	36	lumber disc bulge L4-5, L5-S1	Lumbar Radiculopathy/Lumbar Disc Herniation/ Lumbago With Sciatica
22	44	27	lumber disc bulge L4-5, L5-S1	Lumbar Intervertebral Disc Disorder With Radiculopathy
23	50	28	lumber disc bulge L4-5, L5-S1	Lumbar Radiculopathy/Lumbar Disc Herniation/ Lumbago With Sciatica
24	60	34	lumber spondylosis L4-5, L5-S1	Lumbar Radiculopathy/Lumbar Disc Herniation/ Lumbago With Sciatica

25	57	37	lumber disc bulge L4-5, L5-S1	Acute (Or) Subacute (Or) Chronic Low Back Pain With Radiating Pain
26	42	25	lumber disc bulge L4-5, L5-S1	Acute (Or) Subacute (Or) Chronic Low Back Pain With Radiating Pain
27	51	34	lumber disc bulge L3-4, L4-5, L5-S1	Lumbar Radiculopathy/Lumbar Disc Herniation/ Lumbago With Sciatica
28	35	27	lumber disc bulge L4-5, L5-S1	Lumbar Radiculopathy/Lumbar Disc Herniation/ Lumbago With Sciatica
29	61	34	lumber disc bulge L4-5, L5-S1	Lumbar Radiculopathy/Lumbar Disc Herniation/ Lumbago With Sciatica
30	46	28	lumber disc bulge L4-5, L5-S1	Lumbar Radiculopathy/Lumbar Disc Herniation/ Lumbago With Sciatica
31	55	33	lumber disc bulge L4-5, L5-S1	Lumbar Radiculopathy/Lumbar Disc Herniation/ Lumbago With Sciatica
32	80	29	lumber disc bulge L3-4, L4-5, L5-S1	Lumbar Radiculopathy/Lumbar Disc Herniation/ Lumbago With Sciatica
33	48	29	lumber disc bulge L3-4, L4-5, L5-S1	Lumbar Radiculopathy/Lumbar Disc Herniation/ Lumbago With Sciatica
34	40	26	lumber disc bulge L3-4, L4-5, L5-S1	Lumbar Radiculopathy/Lumbar Disc Herniation/ Lumbago With Sciatica
35	40	24	lumber disc bulge L5-S2	Acute (Or) Subacute (Or) Chronic Low Back Pain With Radiating Pain
36	55	27	lumber disc bulge L3-4, L4-5, L5-S1	Lumbar Radiculopathy/Lumbar Disc Herniation/ Lumbago With Sciatica
37	40	29	lumber disc bulge L4-5, L5-S1	Lumbar Radiculopathy/Lumbar Disc Herniation/ Lumbago With Sciatica
38	50	28	lumber disc bulge L4-5, L5-S1	Lumbar Radiculopathy/Lumbar Disc Herniation/ Lumbago With Sciatica
39	35	29	lumber disc bulge L4-5, L5-S2	Lumbar Radiculopathy/Lumbar Disc Herniation/ Lumbago With Sciatica
40	70	32	lumber disc bulge L4-5, L5-S3	Lumbar Radiculopathy/Lumbar Disc Herniation/ Lumbago With Sciatica
41	46	25	lumber disc bulge L4-5, L5-S4	Lumbar Radiculopathy/Lumbar Disc Herniation/ Lumbago With Sciatica
42	59	24	Multilevel degenerative changes most severe at the level of L5/S1 with diffuse disc bulge and posterior central protrusion	Lumbar Radiculopathy/Lumbar Disc Herniation/ Lumbago with Sciatica
43	56	31	L4-L5 there is diffuse disc bulge impinging the thecal sac and narrowing the lateral recess on both sides	Lumbar Radiculopathy/Lumbar Disc Herniation/ Lumbago with Sciatica
44	64	26	Moderate Multi-level degenerative discs changes of the L3 throughL5 levels with diffuse disk bulge	Lumbar intervertebral disc disorder with radiculopathy

			causing neural foraminal narrowingand stenosis of the lower lumbar spinal cana	
45	39	25	L3-L4 demonstrate minimal posterior diffuse disc bulge indenting the anterior thecal sac and bilateral lateral recess. L4-L5 and L5-S1 demonstrate small posterior diffuse disc bulge that are indenting the anterior thecal sac. No significant neuroforaminal compromise.No disc herniation	Postural Lordosis
46	72	25	Mild lumbar degenerative disc disease	Lumbar Radiculopathy/Lumbar Disc Herniation/ Lumbago with Sciatica
47	38	28	L1-L2: There is diffuse disk bulge multilevel degenerativechanges and disk herniation seen at L2-L3	Acute (or) Subacute Low Back Pain with Mobility Deficits
48	37	27	L4-L5 there is diffuse disc bulge	Lumbar Radiculopathy/Lumbar Disc Herniation/ Lumbago with Sciatica
49	45	29	L3-L4 and L4/L5 there is mild diffuse disc bulge	Lumbar Radiculopathy/Lumbar Disc Herniation/ Lumbago with Sciatic
50	49	39	L4-L5 there is diffuse disc bulge	Lumbar Radiculopathy/Lumbar Disc Herniation/ Lumbago with Sciatica
51	60	28	Multilevel degenerative disk disease	Spinal Instabilities (Dynamic Or Passive), Lumbar Region
52	21	24	diffuse disk bulge and a posterior central protrusion	Derangements Of Sacroiliac Joint/ Sacroiliac Joint Strain/ Sacroiliac Joint Dysfunction/Sacroiliitis
53	60	34	At the level of L2-L3 and L3/L4 there is mild diffuse disc bulge	Lumbar Radiculopathy/Lumbar Disc Herniation/ Lumbago With Sciatica
54	54	25	mild diffuse disk bulge	Spinal Instabilities (Dynamic Or Passive), Lumbar Region
55	60	29	diffuse disc bulge	Lumbar Intervertebral Disc Disorder With Radiculopathy
56	70	34	lumbar degenerative discs disease at the level of L4-L5	Acute (Or) Subacute (Or) Chronic Low Back Pain With Radiating Pain
57	48	21	L4-L5 demonstrates mild diffuse disk bulge	Subacute (Or) Chronic Low Back Pain With Radiating Pain

58	28	23	Minimal degenerative disk disease with mild disk bulge.	Lumbar Radiculopathy/Lumbar Disc Herniation/ Lumbago With Sciatica
59	72	36	t the level of L2-L3, L3-L4, L4-L5 and L5/S1 there is diffuse disc bulge	Lumbar Radiculopathy/Lumbar Disc Herniation/ Lumbago With Sciatica
60	38	26	At the level of L2-L3 there is diffuse disc bulge	Subacute (Or) Chronic Low Back Pain With Radiating Pain
61	56	24	At the level of L3-L4 L4-L5 there is diffuse disc bulge	Lumbar Radiculopathy/Lumbar Disc Herniation/ Lumbago With Sciatica
62	37	32	At the level of L4-L5 there is diffuse disc bulge	Lumbar Radiculopathy/Lumbar Disc Herniation/ Lumbago With Sciatica
63	59	23	At the level of L2-L3 there is mild diffuse disc bulge	Lumbar Radiculopathy/Lumbar Disc Herniation/ Lumbago With Sciatica
64	53	27	severe decrease in the intervertebral disk space involving the L4-L5 level with associated diffuse disk bulge	Lumbar Radiculopathy/Lumbar Disc Herniation/ Lumbago With Sciatica
65	40	32	Degenerated L4-5 and L5-S1 discs	Lumbar Intervertebral Disc Disorder With Radiculopathy
66	50	32	At the level of L3-L4 and L4/L5 there is mild diffuse disc bulge	Lumbar Radiculopathy/Lumbar Disc Herniation/ Lumbago With Sciatica
67	64	30	At the level of L1-L2, L3-L4 and L5-S1 there is mild diffuse disc bulge	Lumbar Radiculopathy/Lumbar Disc Herniation/ Lumbago With Sciatica
68	37	34	No definite evidence of sacroiliitis	Derangement Syndrome With Below Knee Symptoms/Others
69	44	38	L4/L5: diffuse disc bulge	Lumbar Radiculopathy/Lumbar Disc Herniation/ Lumbago With Sciatica
70	65	30	diffuse disc bulge	Lumbar Radiculopathy/Lumbar Disc Herniation/ Lumbago With Sciatica
71	42	37	mild diffuse posterior and posterolateral disc bulges	Lumbar Radiculopathy/Lumbar Disc Herniation/ Lumbago With Sciatica
72	48	31	L3/4 minimal diffuse posterior disc bulge	Lumbar Radiculopathy/Lumbar Disc Herniation/ Lumbago With Sciatica
73	52	26	At the level of L4-L5 there is diffuse disc bulge	Lumbar Discogenic Disease
74	39	22	L5-S1 mild diffuse disc bulge	Lumbar Intervertebral Disc Disorder With Radiculopathy
75	59	30	At the level of L1-L2, L2-L3, L4-L5 there is diffuse disc bulge	Spinal Instabilities (Dynamic Or Passive), Lumbar Region

76	26	24	Minimal posterior diffuse disk bulges	Lumbar Intervertebral Disc Disorder With Radiculopathy
77	57	54	mild diffuse disc bulge	Lumbar Radiculopathy/Lumbar Disc Herniation/ Lumbago With Sciatica
78	57	29	diffuse disc bulge	Lumbar Radiculopathy/Lumbar Disc Herniation/ Lumbago With Sciatica
79	54	27	mild diffuse disc bulge	Lumbar Radiculopathy/Lumbar Disc Herniation/ Lumbago With Sciatica
80	55	38	Mild diffuse disc bulge	Lumbar Radiculopathy/Lumbar Disc Herniation/ Lumbago With Sciatica
81	52	25	level of L3-L4 and L5-S1 there is diffuse disc bulge i	Lumbar Radiculopathy/Lumbar Disc Herniation/ Lumbago With Sciatica
82	67	26	At the level of T12-L1 there is mild central disc bulge	Lumbar Facet Joint Pain/Lumbar Zygapophyseal/Non-Specific Low Back Pain
83	63	27	At the level of L4-L5 there is diffuse disc bulge	Lumbar Radiculopathy/Lumbar Disc Herniation/ Lumbago With Sciatica
84	30	23	At the level of L4-L5 and L5/S1 there is diffuse disc bulge	Lumbar Radiculopathy/Lumbar Disc Herniation/ Lumbago With Sciatica
85	59	22	mild diffuse disk bulge	Lumbar Radiculopathy/Lumbar Disc Herniation/ Lumbago With Sciatica
86	36	33	Minimal Diffuse disc bulge	Lumbar Radiculopathy/Lumbar Disc Herniation/ Lumbago With Sciatica
87	40	21	no significant disc disease	Lumbar Radiculopathy/Lumbar Disc Herniation/ Lumbago With Sciatica
88	38	38	t the level of L4-L5 there is mild diffuse disc bulge	Lumbar Radiculopathy/Lumbar Disc Herniation/ Lumbago With Sciatica
89	33	25	diffuse posterior bulge	Lumbar Radiculopathy/Lumbar Disc Herniation/ Lumbago With Sciatica
90	65	32	minimal diffuse posterior disc bulges	Lumbar Radiculopathy/Lumbar Disc Herniation/ Lumbago With Sciatica
91	50	45	At the level of L3-L4 there is mild diffuse disc bulge	Lumbar Facet Joint Pain/Lumbar Zygapophyseal/Non-Specific Low Back Pain
92	59	21	Lumbar degenerative discs disease at the level of L3-L4 and L4-L5	Spinal Instabilities (Dynamic Or Passive), Lumbar Region
93	25	25	Lumbar degenerative discs disease at the level of L4-L5 and L5-S1 as described	Lumbar Intervertebral Disc Disorder With Radiculopathy
94	44	26	Lumbar degenerative discs disease at the level of L3-L4 and L4-L5 as described	Lumbar Radiculopathy/Lumbar Disc Herniation/ Lumbago With Sciatica

95	41	46	Mild Lumbar degenerative discs disease as described	Lumbar Radiculopathy/Lumbar Disc Herniation/ Lumbago With Sciatica
96	60	28	Multilevel mild degenerative disk disease.Possible irritation of the right S1 nerve root and swelling secondary to the disk protrusion at the level of L5/S1 near the right lateral recess	Osseous And Subluxation Stenosis Of Intervertebral Foramina Of Lumbar Region, Lumbar Region Foraminal Stenosis
97	27	34	Mild Lumbar degenerative discs disease at the level of L4-L5 and L5-S1 with features as described	Lumbar Radiculopathy/Lumbar Disc Herniation/ Lumbago With Sciatica
98	31	32	Lumbar degenerative discs disease at the level of L3-L4, L4-L5 and L5-S1 causing narrowing of the lumbar spinal canal with features as described	Spinal Instabilities (Dynamic Or Passive), Lumbar Region
99	36	34	Lumbar degenerative discs disease at the level of L4-L5 and L5-S1	Lumbar Discogenic Disease
100	53	31	Lumbar degenerative discs disease at the level of L4-L5 and L5-S1	Lumbar Radiculopathy/Lumbar Disc Herniation/ Lumbago With Sciatica
